# Parental employment adjustment during and after childhood cancer treatment — a report from the Swiss Childhood Cancer Survivor Study-Parents

**DOI:** 10.1007/s00520-025-09599-z

**Published:** 2025-06-07

**Authors:** Martina Ospelt, Sonja Kälin, Alexandra Schifferli, André O. von Bueren, Katharina Roser, Gisela Michel

**Affiliations:** 1 Faculty of Health Sciences and Medicine, University of Lucerne, Alpenquai 4, 6005 Lucerne, Switzerland; 2 Department of Hematology/Oncology, University Children’s Hospital Basel, Basel, Switzerland; 3 Department of Pediatrics, Obstetrics and Gynecology Division of Pediatric Hematology and Oncology, University Hospital of Geneva, Geneva, Switzerland; 4 Cansearch Research Platform for Pediatric Oncology and Hematology, Faculty of Medicine, Department of Pediatrics, Gynecology and Obstetrics, University of Geneva, Geneva, Switzerland

**Keywords:** Childhood cancer, Parents, Survivorship, Employment disruptions, Financial burden, Professional burden

## Abstract

**Purpose:**

Parents of children with cancer experience treatment-related employment disruptions. Most significant shifts occur during diagnosis and treatment. However, challenges can persist into survivorship. We explored employment changes during diagnosis and treatment among parents of children with cancer, and professional and financial impact during long-term survivorship. We investigated (1) if and what kind of employment changes occurred, (2) reasons for the changes, (3) differences between mothers and fathers, and (4) if parents experience long-term professional or financial impacts from their child’s past illness.

**Methods:**

A questionnaire survey assessed employment changes during diagnosis and treatment and long-term professional and financial impacts. We described changes and reasons thereof and conducted logistic regression analyses to predict long-term impacts.

**Results:**

We included 469 parents (59% female) of childhood cancer survivors (mean = 24 years after diagnosis). At time of treatment, 21% of parents reported employment changes: e.g., work time reduction (52%), quitting (27%), and taking unpaid leave (21%). Mothers were more likely to experience changes (OR = 2.00; *p* = .005). Most parents (87%), especially mothers, reported caring for their sick child to be one of the reasons for change. Some parents reported professional (5%) or financial long-term impact (5%). Financial impact was mainly associated with survivors experiencing late effects (OR = 10.51; *p* < .001), cancer relapse (OR = 3.96; *p* = .007), and survivors’ financial dependence (OR = 3.64; *p* = .005). Professional impact was associated with female sex (OR = 3.26; *p* = .029) and employment changes (OR = 2.39; *p* = 0.050).

**Conclusions:**

Although most parents do not experience lasting effects on employment or finances, some continue to face challenges well into survivorship. Providing sustained, long-term support for these parents is essential.

**Supplementary Information:**

The online version contains supplementary material available at 10.1007/s00520-025-09599-z.

## Background

The profound disruptions to daily life following a child’s cancer diagnosis present significant challenges for parents. Balancing work, family obligations, and caring for the sick child is demanding [1–5]. Roles and responsibilities for childcare and work have to be reorganized, often resulting in mothers assuming the primary responsibility for the sick child [2–4, 6]. This shift in responsibilities can lead to employment disruptions [7]. A significant number of parents of children with cancer experience treatment-related employment disruptions, including unpaid leave, reduced workloads, alterations to work schedules, or quitting altogether [7–9] with mothers being affected more often [7, 9]. Some studies report up to 94% of families being affected by some sort of employment disruption as a result of their child’s illness [10, 11]. These employment disruptions and thereto related substantial income losses often lead to perceived financial burden for families [7, 10, 11]. Further, the financial burden of childhood cancer is a source of psychosocial stress for parents that may manifest in symptoms such as anxiety, distress, and disrupted sleep [12]. Psychological, familial, and practical factors affect parents’ ability to stay in and/or return to work [4, 5, 13].

Most significant shifts in role dynamics occur during the early stages of diagnosis and initial treatment [2, 4]. However, challenges associated with managing increased caregiving demands and employment disruptions may persist well beyond the end of treatment, extending into the survivorship phase [4, 14]. Many survivors experience various late effects [15, 16], necessitating ongoing support from their family [4], while understanding and support from outsiders diminish over time [13].

Parents serve as the primary informal caregivers for children, and it is crucial to understand their experience of balancing work and parenting, particularly as the demands of caregiving intensify following a cancer diagnosis. However, there has been a lack of studies helping to understand the long-term repercussions that extend well beyond the end of treatment into long-term survival. The aim of this study was to understand employment changes of parents during their child’s cancer diagnosis and treatment in Switzerland, and to examine whether impacts persist into survivorship. Specifically, we investigated (1) if and what kind of employment changes occurred in parents during their child’s cancer diagnosis and treatment, (2) reasons for the employment changes, (3) differences between mothers and fathers, and (4) if parents experience long-term professional or financial impact due to their child’s past illness.

## Methods

### Sample and procedure

This report is part of a larger project investigating psychosocial late outcomes in parents of long-term childhood cancer survivors (CCS) (Swiss Childhood Cancer Survivor Study–Parents (SCCSS–Parents) [17–21]). Parents were recruited through the population-based Swiss Childhood Cancer Registry (SCCR). The SCCR registers all patients with a cancer diagnosis below the age of 20 years in Switzerland. Parents were included if (a) their child was diagnosed with cancer at age ≤ 16 years (1976–2009) according to the International Classification of Childhood Cancer—Third Edition (ICCC-3), (b) their child was a Swiss resident at diagnosis, (c) at least 5 years had passed since diagnosis, and (d) their child was aged ≥ 20 years in 2016 and alive. Eligible parents’ addresses were extracted from the SCCR and verified using the online telephone directory. Parents were then contacted through their formerly treating clinics. First, a study information letter was mailed to all eligible parents. Second, 2 weeks later, parents received two questionnaires (one for each parent) and a prepaid envelope for return. Non-responders were reminded a maximum of two times, 4–6 and 12–14 weeks later (contact period: 01/2017–02/2018). Because parents from all over Switzerland were surveyed, all study documents were available in the three main languages German, French, and Italian.

### Measurements

#### Employment changes and reasons for changes during diagnosis and treatment

Parents reported retrospectively on employment changes during diagnosis and treatment of their child and reasons for employment changes (from a list; supplemental appendix A). For changes and reasons, multiple indications per affected parent were possible. If parents chose “other” for changes or reasons, they were asked to describe in open-ended questions. An overall binary variable for employment changes (yes/no) was coded “yes” if the parent reported any change.

#### Long-term professional impact

Parents were asked about their current professional situation and if it was impacted by their child’s illness (yes/no). Parents were able to explain in what way their employment was still affected in an open-ended question. Additionally, parents were asked whether they were satisfied with their current employment situation (yes/no).

#### Long-term financial impact

Parents were asked whether they were still financially impacted by their child’s illness (yes/no) at time of study; if so, they were asked to describe the nature of these impacts in an open-ended question. They were also asked how well they were able to live on their current household income and if their child is financially independent. A binary variable for CCS’ financial dependence (yes/no) was coded “yes” if parents reported that their child is not or only partially financially independent.

#### Parent characteristics

Parents reported on demographic and socioeconomic characteristics including their sex, age, civil status (coded: single/married/separated/divorced/widowed), household income (coded: 0–3000/3001–4500/4501–6000/6001–9000/9001–12,000/> 12,000), highest educational achievement (coded: compulsory schooling/vocational training/upper secondary education/university education), employment situation (coded: part-time/full-time/retired/homemaker; if, additionally to employment/retirement, homemaker was indicated, employment/retirement was coded), number of children, and parents’ health status (first item of 36-Item Short Form Survey Instrument (SF-36) [22, 23]). A binary variable for parents’ health status (good/poor) was coded “good” when parents indicated excellent, very good or good health, and “poor” when parents indicated fair or poor health.

#### Survivor characteristics

Characteristics describing the survivors, including sex, age at diagnosis, diagnosis (coded: leukemia/lymphoma/CNS tumors/other tumors), and treatment (coded hierarchically: surgery only/chemotherapy (may have had surgery, but not radiotherapy)/radiotherapy (may have had surgery and/or chemotherapy)/stem cell transplantation (may have had surgery and/or chemotherapy and/or radiotherapy)), and relapse (yes/no) were derived from the SCCR. Parents were asked if their child suffers from cancer-related late effects (yes/no).

### Statistical analysis

Statistical analyses were performed using Stata, Version 18 (College Station, TX: StataCorp LLC). To describe the sample, we used descriptive statistics. For aims 1 and 2, descriptive statistics were used to describe whether parents experienced employment changes during the time of diagnosis and treatment, the type of changes, and the reasons for changes. Open-ended responses were used to qualitatively describe “other” changes and reasons.

For aim 3, a mixed-effects logistic regression model was estimated to examine whether response behavior (i.e., one or both parents completed questionnaire) was associated with reported employment changes. The model included a random intercept at the child level and considered the number of responding parents (1 vs. 2) as an independent variable. The association between having both parents respond and reported employment changes was not statistically significant (*β* = − 0.416, 95% CI − 0.956, 0.124, *p* = 0.131). The likelihood ratio test comparing the mixed-effects model to a standard logistic regression indicated that including a random intercept at the child level did not significantly improve model fit (*p* = 0.137). Consequently, subsequent analyses were conducted using standard logistic regressions to test if sociodemographic or survivor characteristics were associated with employment changes. Using chi-square (*χ*^2^) tests, we examined whether employment changes (yes/no), specific types of changes (e.g., reducing work time; yes/no), and reasons for changes (e.g., caring for the sick child; yes/no) differed between mothers and fathers. To examine aim 4 regarding parents’ perceived long-term professional and financial impacts, we first conducted descriptive statistics, followed by logistic regression models to identify factors associated with long-term impacts. To account for potential clustering of responses within families, all models were estimated with robust standard errors clustered at the child level.

All statistically significant variables with *p* < 0.05 in the univariable model were included in the multivariable model for each outcome. We used the open-ended responses to qualitatively describe parents’ experienced long-term impacts. The impacts mentioned were thematically categorized and grouped into overarching themes.

## Results

Parents of 574 eligible CCS were contacted. A total of 478 parents (59.0% mothers) of 308 survivors participated in the questionnaire survey (response rate 53.7%). CCS of participating and non-participating parents were comparable in terms of sociodemographic and cancer-related characteristics. This has been described in more detail elsewhere [24]. Of all participating parents, 469 parents of 303 CCS responded to the questions about employment changes and were included in the analyses. Parental couples made up 336 of these parents. For 133 survivors, only one parent (81.2% mothers) completed the questionnaire. Parents had a mean age of 62.2 years at time of study (SD = 6.8; range 45–85). CCS’ mean age at diagnosis was 6.9 years (SD = 4.6; range 0–15) and mean time since diagnosis was 23.9 years (SD = 7.0; range 7–40). Most parents were married (*n* = 370; 78.9%), had completed vocational training (*n* = 230; 49.0%), and were employed part-time (*n* = 139; 29.6%) or full-time (*n* = 116; 24.7%) at time of study. Around a third (*n* = 159; 33.9%) were retired (Table 1).

**Table 1:** Characteristics of participating parents and their children who survived childhood cancer

Characteristics of parents (*N* = 469)								
		Mean		SD		Range	*N*	%
Age at study (years)	Overall Mothers Fathers	62.2 61.3 63.4		6.8 6.7 6.7		45–85 45–85 49–83		
Age at diagnosis (years)	Overall Mothers Fathers	38.4 37.3 39.9		6.3 5.9 6.5		21–64 21–57 23–64		
Sex	Female Male						276 193	58.9 41.1
Civil status at study	Single Married Separated Divorced Widowed						5 370 4 37 25	1.1 78.9 0.9 7.9 5.3
Number of children at study	1 2 3 4 ≥ 5						7 208 132 52 30	1.5 44.4 28.1 11.1 6.4
Educational achievement at study	Compulsory schooling Vocational training Upper secondary education University education						52 230 77 69	11.1 49.0 16.4 14.7
Employment situation at study	Employed full time *Mothers* *Fathers* Employed part time *Mothers* *Fathers* Homemaker *Mothers* *Fathers* Retired *Mothers* *Fathers*						116 *29* *87* 139 *117* *22* 39 *38* *1* 159 *80* *79*	24.7 *25.0* *75.0* 29.6 *84.2* *15.8* 8.3 *97.4* *2.56* 33.9 *50.3* *49.7*
Household income (in CHF per month) at study	0–3000 3001–4500 4501–6000 6001–9000 9001–12,000 > 12,000						31 32 80 128 64 67	6.6 6.8 17.1 27.3 13.7 14.3
Health status at study	Excellent						64	13.7
	Very good						190	40.5
	Good						190	40.5
	Fair						16	3.4
	Poor						5	1.1
**Characteristics of survivors (*N* = 303)**								
	Mean		SD		Range		*N*	%
Age at study (years)	32.2		6.3		21–54			
Age at diagnosis (years)	6.9		4.6		0–15			
Time since diagnosis (years)	23.9		7.0		7–40			
Sex	Female Male						137 166	45.2 54.8
Diagnosis	Leukemia Lymphoma CNS tumor Neuroblastoma Retinoblastoma Renal tumor Hepatic tumor Malignant bone tumor Soft tissue sarcoma Germ cell tumor Langerhans cell histiocytosis						105 52 36 13 9 20 6 15 23 10 11	34.7 17.2 11.9 4.3 3.0 6.6 2.0 5.0 7.6 3.3 4.6
Treatment	Surgery Chemotherapy Radiotherapy Stem cell transplantation						37 168 78 19	12.3 55.6 25.8 6.3
Relapse	Yes No						38 265	12.5 87.5
Late effects (parent-reported)	Yes No						116 177	38.3 58.4

*CNS* central nervous system.

Observations may not sum to total *N* due to missing values. Percentages are based on the total number of participants and may not add up to 100 due to missing values.

### Prevalence of employment changes

In total, 20.9% (*n* = 98) of parents indicated employment changes at the time of their child’s diagnosis and treatment. Mothers (*n* = 70; 71.4%) were more likely than fathers (*n* = 28; 28.6%; *χ*^2^ = 8.10;* p* = 0.004; Table 2) to report changes. We did not find any other significant association between employment changes and demographic or diagnosis- and treatment-related characteristics (supplemental appendix B).

**Table 2:** Chi-square (*χ*^2^) tests investigating differences between mothers and fathers for employment changes and reasons

	Chi-square (*χ*^2^) tests		
	“Yes” reported by mothers (*N* = 276) *n* (%)	“Yes” reported by fathers (*N* = 193) *n* (%)	*p*-value
**Employment changes (*N* = 469)**			
Employment change (yes/no)	70 (25.4)	28 (14.5)	**0.004**
**Types of employment changes**			
Work time reduction (yes/no)	33 (47.1)	17 (60.7)	0.250
Quitting job (yes/no)	23 (33.3)	3 (10.1)	**0.023**
Unpaid leave (yes/no)	13 (18.5)	7 (25.0)	0.497
Changing job (yes/no)	3 (4.3)	2 (7.1)	0.573
Work time increase (yes/no)	1 (1.4)	2 (7.1)	0.142
Job loss (yes/no)	0 (0.0)	1 (3.6)	0.115
**Reasons for employment changes**			
Caring for the sick child (yes/no)	58 (82.9)	18 (64.3)	0.126
Too little time for other family members (yes/no)	22 (31.4)	11 (39.3)	0.254
Job not being a priority (yes/no)	21 (30.0)	1 (3.6)	**0.007**
Being exhausted (yes/no)	17 (24.3)	0 (0.0)	**0.006**
Shift in interest (yes/no)	1 (1.4)	1 (3.6)	0.445
Financial reasons (yes/no)	0 (0.0)	1 (3.6)	0.093

Bold font indicates statistically significant results.

Multiple indications per affected parent for changes and reasons possible.

**Fig. 1:**
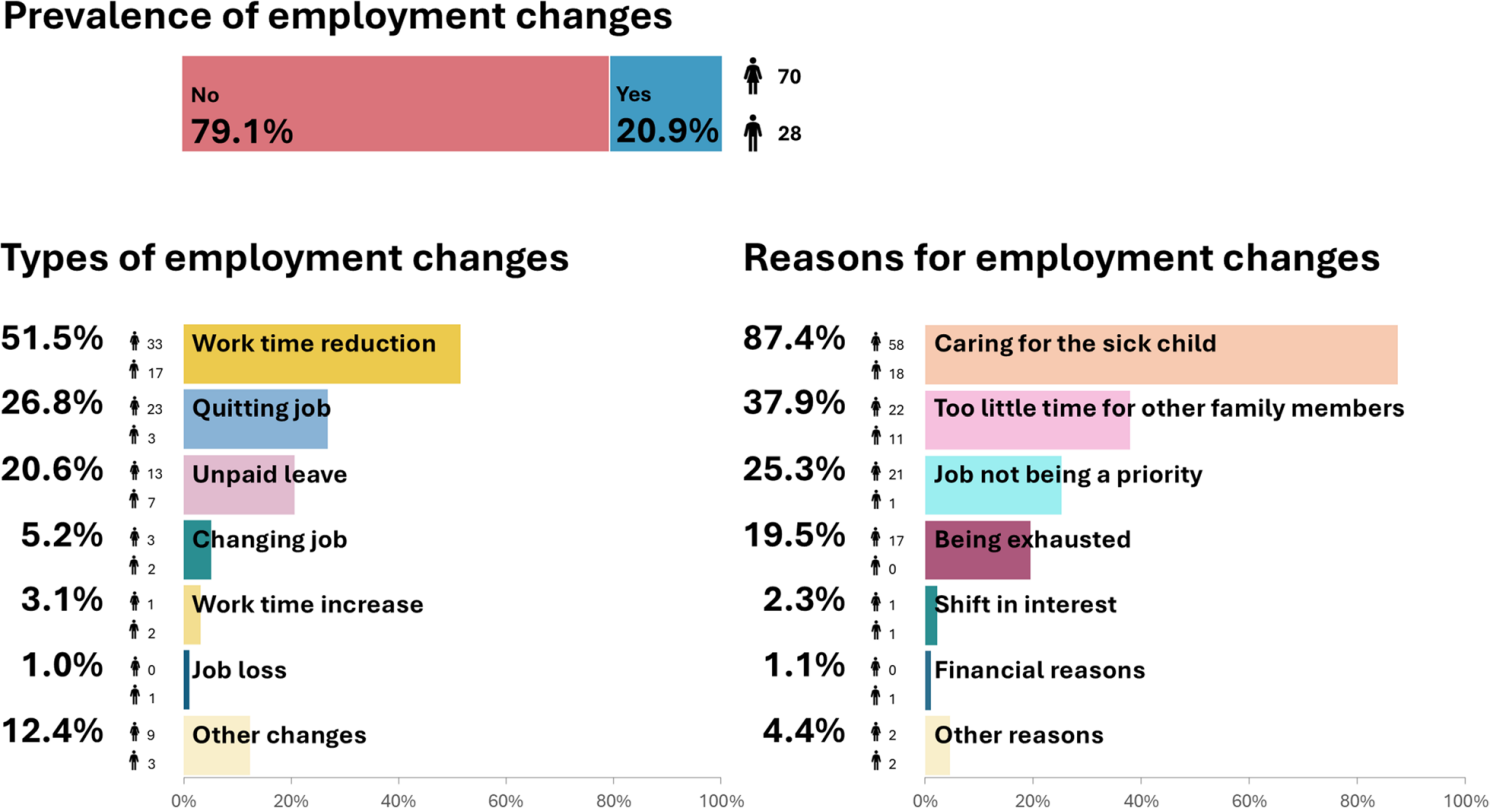
Parental employment changes and reasons for employment changes. Note: Multiple indications per affected parent possible. Data for mothers and fathers in absolute numbers

Among parents with employment changes, the most frequently reported changes were as follows: reducing work time (*n* = 50; 51.5%), quitting job (*n* = 26; 26.8%), and taking unpaid leave (*n* = 20; 20.6%). Some parents reported changing jobs (*n* = 5, 5.2%), increasing work time (*n* = 3; 3.1%), or losing their job (*n* = 1; 1.0%) during the time of diagnosis and treatment (Fig. 1). Mothers (*n* = 23; 88%) were more likely to quit their job than fathers (*n* = 3; 12%; *χ*^2^ = 5.19, *p* = 0.023; Table 2). Regarding the types of employment changes, this was the only significant difference identified between mothers and fathers. Furthermore, 12 parents (12.4%) indicated they had experienced other types of employment changes, such as reducing international travel for work or shifting working hours from daytime to evenings/weekends to avoid falling behind and/or losing pay.

In addition to the 98 parents who reported employment changes, 13 mothers who were not employed at the time of diagnosis explicitly responded to open-ended questions that although they did not experience employment changes, their workload at home had changed with increased care-taking responsibilities.

### Reasons for employment changes

Of the 98 parents who reported employment changes, 87 provided reasons for the changes. Caring for the sick child was the most frequently reported reason, reported by 76 parents (87.4% of parents with employment changes). More than a third of affected parents (*n* = 33; 37.9%) reported having too little time for other family members and 25.3% (*n* = 22) indicated the job not being a priority as reasons for employment changes. Changes because of being exhausted were reported by 19.5% (*n* = 17) of parents. Few reported a shift in interest (*n* = 2; 2.3%), financial aspects (*n* = 1; 1.1%), and “other” (*n* = 4; 4.6%). Mothers were more likely than fathers to report the job not being a priority (*n* = 21; 95.5% vs. *n* = 1; 4.5%; *χ*^2^ = 8.18, *p* = 0.007; Table 2) and being exhausted (*n* = 17; 100% vs. *n* = 0; 0%; *χ*^2^ = 8.37, *p* = 0.006) as reasons (Fig. 1).

### Long-term impact

#### Long-term professional impact

**Fig. 2:**
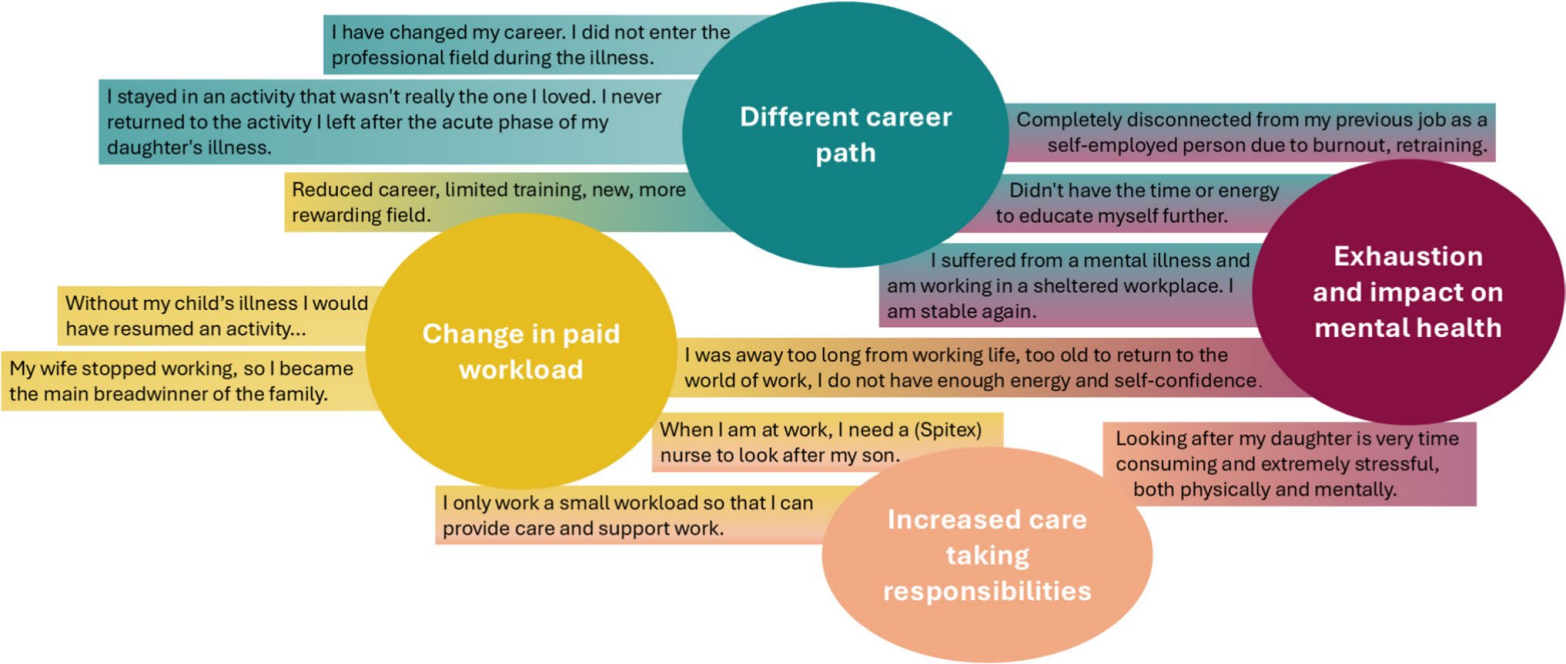
Impact of the child’s illness on parents’ current employment situation. Example quotes from open-ended questions

At time of survey, 21 parents (4.5%) reported still being professionally impacted by their child’s cancer. On average, this was 21.6 years after the cancer diagnosis (SD = 5.8; range 7–31). Overarching themes such as changes in (paid) workload due to increased care taking responsibilities, exhaustion, or following a different career path after their child’s cancer were identified as impacts (Fig. 2). Most parents (*n* = 400; 85.3%), however, reported satisfaction with their current employment situation.

Long-term professional impact was significantly associated with female sex (OR = 2.98, *p* = 0.048; Table 3) in multivariable analyses and with employment changes at time of diagnosis and treatment (OR = 2.39, *p* = 0.050) in univariable analysis.

**Table 3:** Univariable and multivariable logistic regressions investigating the factors associated with perceived long-time professional and financial impact

	Univariable logistic regressions								Multivariable logistic regressions							
	Professional impact				Financial impact				Professional impact				Financial impact			
	OR	95% CI	*p*-value	Sample size	OR	95% CI	*p*-value	Sample size	OR	95% CI	*p*-value	Sample size	OR	95% CI	*p*-value	Sample size
Parent characteristics																
Sex				*N* = 422				*N* = 461								
Male	ref				ref											
Female	3.258	[1.127, 9.417]	**0.029**		1.085	[0.495, 2.377]	0.838		2.982	[1.010, 8.809]	**0.048**	*N* = 422				
Age at diagnosis (years)	1.002	[0.918, 1.093]	0.966	*N* = 418	1.038	[0.976, 1.100]	0.235	*N* = 457								
Age at study (years)	0.961	[0.887, 1.042]	0.338	*N* = 418	1.026	[0.971, 1.084]	0.369	*N* = 457								
Employment change (yes)	2.394	[0.999, 5.738]	**0.050**	*N* = 422	2.062	[0.856, 4.970]	0.107	*N* = 461	2.096	[0.858, 5.112]	0.104	*N* = 422				
Employment situation at study				*N* = 417				*N* = 447								
Employed full time	ref				ref											
Employed part time	2.309	[0.840, 6.346]	0.105		1.177	[0.455, 3.048]	0.737									
Homemaker	0.716	[0.081, 6.362]	0.764		1.959	[0.448, 8.567]	0.372									
Retired	0.344	[0.065, 1.834]	0.212		1.208	[0.384, 3.803]	0.747									
Education at study				*N* = 388				*N* = 421								
Compulsory schooling	ref				ref											
Vocational training	1.568	[0.334,7.363]	0.569		1.367	[0.289, 6.465]	0.693									
Upper secondary education	0.303	[0.026, 3.474]	0.337		2.130	[0.408, 11.130]	0.370									
University education	1.410	[0.245, 8.104]	0.700		0.366	[0.031, 4.249]	0.421									
Health status				*N* = 419				*N* = 458								
Good	ref				ref											
Poor	2.359	[0.505, 11.015]	0.275		3.475	[0.914, 13.208]	0.067									
Survivor characteristics																
CCS’ financial dependence (yes)	2.054	[0.803, 5.250]	0.133	*N* = 421	3.640	[1.474, 8.991]	**0.005**	*N* = 460					3.193	[1.221, 8.353]	**0.018**	*N* = 446
CCS’ age at diagnosis (years)	0.962	[0.866, 1.068]	0.463	*N* = 422	1.013	[0.932, 1.101]	0.758	*N* = 461								
Time since diagnosis (years)	0.962	[0.907, 1.021]	0.205	*N* = 422	0.986	[0.927, 1.050]	0.665	*N* = 461								
Diagnosis				*N* = 422				*N* = 461								
Leukemia	ref				ref											
Lymphoma	1.180	[0.361, 3.859]	0.785		0.703	[0.214, 2.317]	0.563									
CNS tumor	1.069	[0.260, 4.402]	0.926		0.252	[0.031, 2.067]	0.199									
Other tumor	0.562	[0.170, 1.856]	0.345		0.577	[0.195, 1.714]	0.323									
Treatment				*N* = 421				*N* = 459								
Surgery	ref				ref											
Chemotherapy	0.830	[0.221, 3.120]	0.782		2.418	[0.293, 19.951]	0.412									
Radiotherapy	0.962	[0.221, 4.192]	0.959		4.252	[0.498, 36.275]	0.186									
Stem cell transplantation	2.545	[0.366, 17.690]	0.345		10.727	[1.117, 103.021]	**0.040**						3.280	[0.246, 43.926]	0.369	*N* = 446
Late effects (yes)	1.937	[0.760, 4.936]	0.166	*N* = 411	10.505	[2.988, 36.935]	**0.000**	*N* = 448					8.248	[2.102, 32.359]	**0.002**	*N* = 446
Relapse (yes)	1.772	[0.572, 5.490]	0.321	*N* = 422	3.960	[1.460, 10.734]	**0.007**	*N* = 461					1.802	[0.587, 5.530]	0.303	*N* = 446

*CCS* childhood cancer survivor, *CI* confidence interval, *CNS* central nervous system, *OR* odds ratio, *ref* reference category.

Bold font indicates statistically significant results.

#### Long-term financial impact

At time of survey, 23 (4.9%) parents reported still being financially impacted due to their child’s cancer. On average, this was 23.0 years after cancer diagnosis (SD = 6.7; range 7–36). When asked in what respect they were still impacted, most parents (*n* = 14) reported financially supporting their child who survived cancer. Some provided occasional financial support like paying for education, holidays, transportation, or hobbies, and others supported their child with monthly payments or covering living and health insurance expenses. Some parents (*n* = 4) reported still paying off significant costs that arose from their child’s illness. Two mothers reported financial repercussions on their pension funds.

Still experiencing financial impact was significantly associated with having a child who was financially dependent (OR = 3.19, *p* = 0.018; Table 3) and having a child suffering from late effects (OR = 8.25, *p* = 0.002) in multivariable analyses. Having a child who had relapsed (OR = 3.96, *p* = 0.007) and having a child who received stem cell transplantation (vs. surgery; OR = 10.73, *p* = 0.040) was significantly associated with financial impact in univariable analysis, only. When asked about how well parents were able to live on their current income, 67.6% (*n* = 317) indicated they could live well, 23.9% (*n* = 112) indicated it was just enough, and 4.1% (*n* = 19) indicated it was difficult or very difficult to make ends meets. Only three parents reported both professional and financial impact at time of survey. More than one fourth (*n* = 128; 27.3%) reported that their formerly sick child was not financially independent at time of study.

## Discussion

The aim of this study was to better understand parents’ employment changes during diagnosis and treatment of their child’s cancer in Switzerland, and to examine whether impacts persist into survivorship. We found that one-fifth of parents experienced employment changes during their child’s cancer diagnosis and treatment. Mothers were more often affected than fathers. Most frequently reported changes were work time reduction, reported by over half of affected parents, followed by a quarter who reported quitting and a fifth who reported taking unpaid leave. Most parents, especially mothers, reported caring for their sick child to be a reason for change. Some parents, especially mothers and parents with children suffering from long-term effects, reported professional or financial impacts long into survivorship, on average more than 20 years after their child’s diagnosis.

Childhood cancer has a profound impact on parents’, particularly mothers’, employment situation [7]. In line with our findings, a systematic review of international literature shows that unfavorable socio-economic consequences were most imminent at time of diagnosis and treatment, but for some parents persisted into survivorship [7]. The present study, based on a sample of parents of long-term CCS, demonstrates how initial repercussions can affect families’ financial and professional situations long after cancer treatment ended.

In previous research, two thirds of parents reported employment disruptions during their child’s cancer treatment [4, 11]. Although the overall proportion of parents experiencing changes was considerably lower in our study, the patterns of changes observed align with previous findings [11]. Regarding the three most frequently reported employment changes, namely work time reduction, quitting, and unpaid leave, the proportions we found were similar [11].

A potential explanation for identifying fewer employment disruptions overall compared to other reports [4, 11, 25] may be attributed to the more dominant traditional family roles in Switzerland on average 24 years ago. According to the Swiss Federal Statistical Office (BFS), the proportion of mothers who are not employed has declined since 2010. For the year 2023, the BFS reported that approximately 80% of fathers and 20% of mothers with children under the age of 25 and a partner were employed in full-time positions. Consequently, most employed mothers (approximately 60%) and a minority of employed fathers (approximately 15%) were engaged in part-time work. In 2023, fathers were employed in full-time positions slightly less frequently than in 2010, while part-time employment had increased [26]. For our population, this implies that fathers would typically be employed while mothers were either homemakers or working part time, a situation that would persist throughout their child’s diagnosis and treatment. During that period, mothers assumed greater caregiving responsibilities but did not experience changes in their employment status. This enabled fathers to maintain full-time employment and avoid employment changes. Another Swiss study that examined the long-term employment situation (mean time since diagnosis 9 years) of parents of CCS (mean age at study 12 years) found that in 2010/2011 approximately one-third of participating mothers were not employed, whereas over 90% of fathers were employed full-time [14].

Interestingly, while mothers were significantly more likely than fathers to report overall employment changes, the only specific difference was that mothers were more likely to quit their jobs. In line with this, more mothers reported caring for their sick child to be a reason for employment changes. Mothers were also significantly more likely to report the job not being a priority, and being exhausted as reasons for changes, making quitting a logical consequence. It has been shown that a significant proportion of care-taking responsibilities falls on mothers of children with cancer [2–4, 6]. In circumstances where one partner is obliged to relinquish their employment, and the family must rely on a single income, financial considerations are also relevant. This may also explain why mothers are more likely to give up paid work [27].

Reducing (paid) workload or withdrawing from working life increases the likelihood of unsuccessful reintegration into the workforce [4, 11]. In an Australian study, on average 2 years after treatment completion, two thirds of parents reported ongoing employment disruptions and barriers to reentering the workforce or increasing working hours [11]. In our study, some mothers explicitly articulated in open-ended questions that they had either reduced their workload or not resumed their paid work to continue caring for their child and expressed the notion that full reintegration into the workforce was not a viable option, which in turn resulted in perceived long-term professional impact.

In terms of long-term impacts, we found that, on average, more than 20 years after diagnosis, some parents reported that their child’s cancer was still affecting them financially or professionally. Mothers were more prone to experience current professional impacts compared to fathers. Parents who had experienced employment changes at time of diagnosis and treatment were also more likely to report long-term professional impacts compared to those who had not. Associated with long-term financial impact was having a child suffering from late effects, having relapsed from cancer or not being financially independent. In addition, parents with poorer health tended to be more likely to report financial impacts. Parents who provide long-term care and support for their adversely affected children seem to be more susceptible to experiencing negative impacts themselves. Interestingly, most parents experienced either long-term professional or financial impact, but rarely both. However, the proportion of parents reporting long-lasting impacts was rather small. This suggests that most parents will recover in these aspects of their lives.

### Implications

The burden of a child’s illness affects parents in various ways. Providing appropriate support is crucial. In addition to potentially expanding existing support services, we believe it is essential to consider employer- and government-based solutions. For instance, workplace characteristics, such as flexibility and support, play a significant role in parents’ ability to balance paid employment with unpaid responsibilities, like caring for a sick child [4, 11, 13, 28]. The wider implementation of flexible and remote working hours, or compressed work weeks models, would allow parents to select work schedules that align with personal commitments, including hospital visits, caregiving responsibilities, and domestic tasks. This approach would enable parents to care for their children while remaining in the labor market, benefiting not only parents of chronically ill children but working parents in general.

Another approach may be expanding options for care leave without parents sacrificing their job security. Parents in our study were only entitled to 3 days of paid care leave under the Swiss Code of Obligations [29]. Only as of 2021, under the Income Compensation Ordinance (EO), working parents in Switzerland can claim up to 14 weeks of care leave with a daily allowance equal to 80% of their salary to care for children with severe health impairments [29, 30]. Given the complex and treatment-intensive nature of an illness such as childhood cancer, 14 weeks may be insufficient. However, such regulations provide some relief from the burden of balancing work and care.

Family caregivers are the backbone of unpaid home care [31–33]. Parents of children with cancer provide care and support in a variety of ways throughout the treatment phase and into survivorship. Innovative models for employing family caregivers through home care agencies have been proposed in Switzerland and the US as potential solutions to help “lighten the load” [31]. The caregiver’s salary is funded through payer’s reimbursement (in the US, Medicaid; in Switzerland, a combination of private health insurance and the municipality). Such models could, at the very least, serve to alleviate the financial burden by ensuring that family caregivers are not only compensated for their care work but also protected by an employment contract regulating working hours, training and benefits like holidays, sick leave, and contributions to pension and social security funds. Although first introduced in a few Swiss municipalities in 2000, research on the model is scarce and it has so far focused primarily on geriatric care [31]. Nevertheless, we believe that considering and further testing such models for different circumstances could be a step towards addressing the double burden borne by parents balancing caregiving and other responsibilities.

### Limitations

Our study is based on retrospective questionnaire data; thus, our results are constrained by general limitations such as recall bias and more general to questionnaire studies, non-response bias. However, a major strength of this study is that we were able to assess long-term impacts of childhood cancer on the employment and financial situation of parents and gain insight into significant employment changes and reasons thereof. Our population-based cohort sample of parents, including many fathers, is another strength of this study.

## Conclusion

Our study showed that many parents experience employment changes throughout their child’s cancer treatment—especially mothers. The change in role towards the care of the sick child was the main reason for the changes. While most parents do not face lasting setbacks in employment and finances, some continue to struggle long after treatment. Survivors’ long-term effects were identified as one of the main determinants for parents’ perceived current burden. This underscores the necessity for short- and long-term sustained support for parents.

## Supplementary Information

Below is the link to the electronic supplementary material.

Supplementary file1 (PDF 305 KB)

## Data Availability

Requests for data from the Swiss Childhood Cancer Survivor Study–Parents (SCCSS-Parents) should be addressed to the corresponding author. Individual-level, fully anonymized, sensitive data can only be made available to researchers who meet the respective legal requirements. Requests for data from the Childhood Cancer Registry must be directed to the Childhood Cancer Registry of Switzerland (https://www.childhoodcancerregistry.ch/).
