# Supplementary material for: Parental employment adjustment during and after childhood cancer treatment — a report from the Swiss Childhood Cancer Survivor Study-Parents
Source: Support Care Cancer. 2025 Jun 7;33(7):556. doi: 10.1007/s00520-025-09599-z (PMC12145287; doi:10.1007/s00520-025-09599-z)
Supplement: Supplementary file 1 — Supplementary file1 (PDF 305 KB) [file 520_2025_9599_MOESM1_ESM.pdf]

Appendix to the manuscript [Supportive Care in Cancer]

Parental employment adjustment during and after childhood cancer treatment – a report from the Swiss Childhood Cancer Survivor Study-Parents

Martina Ospelt[1], Sonja Kälin[1], Alexandra Schifferli[2], André O. von Bueren[3,4], Katharina Roser[1*], Gisela Michel[1*]

[1] University of Lucerne, Faculty of Health Sciences and Medicine, Lucerne, Switzerland

[2] Department of Hematology/Oncology, University Children's Hospital Basel, Basel, Switzerland

[3] Department of Pediatrics, Obstetrics and Gynecology Division of Pediatric Hematology and Oncology University Hospital of Geneva, Geneva, Switzerland

[4] Cansearch Research Platform for Pediatric Oncology and Hematology, Faculty of Medicine, Department of Pediatrics, Gynecology and Obstetrics, University of Geneva, Geneva, Switzerland

[*] shared last authorship

Corresponding author: Gisela Michel, Faculty of Health Sciences and Medicine, University of Lucerne, Alpenquai 4, 6005 Lucerne, Switzerland; gisela.michel@unilu.ch

Overview:

Supplemental Appendix A – Excerpt from the parent questionnaire

Supplemental Appendix B – Univariable logistic regressions investigating the factors associated with employment changes

Supplemental appendix A – Excerpt from the parent questionnaire

(translated from original German questionnaire)

Employment situation, employment changes and reasons for changes during diagnosis and treatment

Did your employment situation change during the diagnosis and treatment of your child?

- ☐ No → *if no, got to question X*
- ☐ Yes

How did your employment situation change **at the time**? (multiple answers possible)

- ☐ Gave up work voluntarily (resignation)
- ☐ Loss of job (dismissal by employer)
- ☐ Reduced hours
- ☐ Increased hours
- ☐ Unpaid leave
- ☐ Took a second job
- ☐ Changed jobs
- ☐ Other: ✎ ____

____

____

What were your reasons **at the time** for changing your employment situation?
(multiple answers possible)

- ☐ Change of interest / career (positive aspects)
- ☐ Job not a priority
- ☐ Time required to care for the sick child
- ☐ No energy (physical / mental exhaustion)
- ☐ Not enough time left for family members other than the sick child
- ☐ Financial aspects
- ☐ Other: ✎ ____

____

____

What is your current employment situation on the labor market?
(multiple answers possible)

	Number of hours per week:
☐ One job (full-time)	_______ hours
☐ One job (part-time, at least 1 hour per week)	_______ hours
☐ Multiple jobs (part-time)	_______ hours
☐ Looking for a job (registered with the unemployment insurance or not)	
☐ In training (school, studies, apprenticeship)	
☐ Homemaker	
☐ Disabled or partially disabled person (e.g. IV pensioner)	
☐ Retired (AHV, other pension) or pensioner except disability pensions	
☐ Other situation without employment	

Are you satisfied with your **current** job situation?

☐ No
☐ Yes

Is your **current** job situation affected by your child's illness?

☐ No
☐ Yes

If yes, please briefly describe how your child's illness affects your current job situation.

____

____

____

Questions about your **vocational training**. Please list **all** the educational training you have completed in column a) and your current training in column b).
(multiple answers possible)

a) **Completed**	b) Currently **in training**	
☐	☐	None
☐	☐	Up to a maximum of 7 years of compulsory schooling
☐	☐	Compulsory schooling (pre-higher secondary school section, general section, basic section, special school)
☐	☐	1-year pre-apprenticeship, 1-year general education school, 10th school year, 1-year vocational orientation school, household service year, language school (at least 1 year) with final certificate, social year, bridge year or similar training
☐	☐	2- to 3-year diploma middle school, traffic school, specialized middle school FMS or similar training
☐	☐	Basic vocational training (apprenticeship, 2- to 4-year vocational apprenticeship or full-time vocational school, commercial diploma, training workshop or similar training)
☐	☐	Baccalaureate, teacher training seminar (preparatory training for kindergarten and primary school teachers, handicrafts, home economics)
☐	☐	Federal vocational or specialized baccalaureate
☐	☐	Higher vocational training with a federal specialist certificate, federal diploma or master's diploma, higher technical college for technology (TS), for economics (HKG) or similar higher technical college (2 years full-time or 3 years part-time study)
☐	☐	Higher technical college (predecessor of universities of applied sciences, e.g. HTL, HWV, HFG, HFS) including post-graduate diploma (3 years full-time or 4 years part-time study)
☐	☐	Bachelor's degree (university, ETH, university of applied sciences, university of teacher education)
☐	☐	Master's degree, licentiate, diploma, state examination, post-graduate diploma (university, ETH, university of applied sciences, university of teacher education)
☐	☐	Doctorate, habilitation

Please indicate approximately your **monthly net income**, i.e. the income after deduction of compulsory social security contributions and pension fund contributions.

Income in CHF

☐ Not employed
☐ 0-3000 Fr.
☐ 3001-4500 Fr.
☐ 4501-6000 Fr.
☐ 6001-9000 Fr.
☐ 9001-12000 Fr.
☐ More than 12000 Fr.

Please give a rough estimate of the total **monthly net income of your household**.
Household income in Swiss francs: This is the sum of all the incomes of all household members added together, after deduction of compulsory social security contributions and pension fund contributions, plus, or minus any alimony.

☐ 0-3000 Fr.
☐ 3001-4500 Fr.
☐ 4501-6000 Fr.
☐ 6001-9000 Fr.
☐ 9001-12000 Fr.
☐ More than 12000 Fr.

How well do you live on your current household income?
(Please tick the answer that best applies to you)

☐ It is easy to live on the current income
☐ The current income is just enough
☐ It is difficult to make ends meet on the current income
☐ It is very difficult to make ends meet on the current income

Are you still financially burdened by your child's illness **today**?

☐ No
☐ Yes

If yes, how / by which means?

____

____

____

Is your child financially independent?

- ☐ Yes
- ☐ Partially
- ☐ No

Does your child suffer from long-term effects of cancer?

- ☐ No
- ☐ Yes

If yes, which ones?

____

____

____

In general, how would you describe your state of health?

Excellent	☐	1
Very good	☐	2
Good	☐	3
Fair	☐	4
Poor	☐	5

Personal details

Please answer the following questions about yourself:

What is your date of birth?

Day __________ Month __________ Year __________

Sex

☐ Female ☐ Male

What is your civil status?

- ☐ Single
- ☐ Married Since ____________ (year)
- ☐ Widowed Since ____________ (year)
- ☐ Divorced Since ____________ (year)
- ☐ Separated Since ____________ (year)

Supplemental appendix B – Univariable logistic regressions investigating the factors associated with employment changes

Table A1: Univariable logistic regressions investigating the factors associated with employment changes

	Employment changes			
	OR	95% CI	p-value	Sample size
Parent characteristics				
Sex	2.002	[1.234, 3.248]	**0.005**	N = 469
(Ref Male)				
Age at diagnosis (years)	1.016	[0.980, 1.052]	0.390	N = 465
Education				N = 428
(Ref Compulsory schooling)				
Vocational training	0.884	[0.410, 1.908]	0.754	
Upper secondary education	1.102	[0.456, 2.662]	0.830	
University education	1.838	[0.778, 4.340]	0.165	
Survivor characteristics				
Sex	1.129	[0.722, 1.764]	0.595	N = 469
(Ref Male)				
CCS' age at diagnosis (years)	1.013	[0.964, 1.064]	0.610	N = 469
Diagnosis				N = 469
(Ref Leukemia)				
Lymphomas	0.558	[0.267, 1.165]	0.120	
CNS tumors	1.436	[0.720, 2.862]	0.304	
Other tumors	0.894	[0.529, 1.511]	0.676	
Treatment				N = 467
(Ref Surgery)				
Chemotherapy	1.137	[0.538, 2.403]	0.736	
Radiotherapy	1.932	[0.880, 4.239]	0.101	
Stem cell transplantation	1.190	[0.363, 3.907]	0.774	

Abbreviations: CCS = childhood cancer survivor, CI = confidence interval, CNS = central nervous system, OR = odds ratio, Ref = reference category
Note: Bold font indicates statistically significant results.
